# Decitabine Promotes Modulation in Phenotype and Function of Monocytes and Macrophages That Drive Immune Response Regulation

**DOI:** 10.3390/cells10040868

**Published:** 2021-04-12

**Authors:** Fabiana Albani Zambuzi, Priscilla Mariane Cardoso-Silva, Ricardo Cardoso Castro, Caroline Fontanari, Flavio da Silva Emery, Fabiani Gai Frantz

**Affiliations:** 1 Faculdade de Ciências Farmacêuticas de Ribeirão Preto–FCFRP-USP, Ribeirão Preto, São Paulo 14041-903, Brazil; fabi_zambuzi@hotmail.com (F.A.Z.); priscillamarianecs@gmail.com (P.M.C.-S.); ricardoccastro@usp.br (R.C.C.); fontanari@usp.br (C.F.); flavioemery@usp.br (F.d.S.E.); 2 Faculdade de Ciências da Saúde de Barretos Dr. Paulo Prata–FACISB, Barretos, São Paulo 14785-002, Brazil

**Keywords:** DNMT inhibitors, phagocytosis, immune cell, monocyte/macrophages

## Abstract

Decitabine is an approved hypomethylating agent used for treating hematological malignancies. Although decitabine targets altered cells, epidrugs can trigger immunomodulatory effects, reinforcing the hypothesis of immunoregulation in treated patients. We therefore aimed to evaluate the impact of decitabine treatment on the phenotype and functions of monocytes and macrophages, which are pivotal cells of the innate immunity system. In vitro decitabine administration increased bacterial phagocytosis and IL-8 release, but impaired microbicidal activity of monocytes. In addition, during monocyte-to-macrophage differentiation, treatment promoted the M2-like profile, with increased expression of CD206 and ALOX15. Macrophages also demonstrated reduced infection control when exposed to *Mycobacterium tuberculosis* in vitro. However, cytokine production remained unchanged, indicating an atypical M2 macrophage. Furthermore, when macrophages were cocultured with lymphocytes, decitabine induced a reduction in the release of inflammatory cytokines such as IL-1β, TNF-α, and IFN-γ, maintaining IL-10 production, suggesting that decitabine could potentialize M2 polarization and might be considered as a therapeutic against the exacerbated immune response.

## 1. Introduction

Monocytes and macrophages are crucial cells involved in the immune response, playing an important role in infectious diseases, tumor immune response, tissue repair, and homeostasis maintenance, through their ability to phagocytose and release immune mediators that coordinate inflammatory responses and drive adaptive immunity [1]. In the presence of an inflammatory stimulus in the tissue compartment, monocytes are recruited to inflammatory foci and differentiate into macrophages based on microenvironmental conditions. Based on the proportion of cytokines, chemokines, and growth factors produced during pathological processes, monocytes can be differentiated into two major groups of macrophages, with different functions in the immune response [2,3].

Among these subpopulations, M1 macrophages are described as proinflammatory cells, with killing effects on pathogens and tumor cells and proinflammatory cytokine production, being involved in inflammatory diseases [4,5] This type of cell is induced by inflammatory stimuli such as interferon-gamma (IFN-γ) and granulocyte-macrophage colony-stimulating factor (GM-CSF) produced by other immune cells or bacterial products such as lipopolysaccharides (LPS) [2,3]. On the other hand, M2 macrophages are related to tumor progression, parasite infections, allergic diseases, and tissue remodeling, and in producing IL-10 and TGF-β. This polarization pattern could be driven by IL-4, IL-13, and IL-10 [3,6,7]. Despite these two extreme phenotypes, a wide range of macrophage subpopulations, such as M2a, M2b, and M2c, that act in different diseases and microenvironment contexts, are also observed [8].

It has already been described that epigenetic modifications are directly related to cell polarization, as demonstrated by T helper cells [9,10] and macrophages M1 and M2 profiles [11,12]. Among the epigenetic changes, DNA methylation patterns and DNA methyltransferase (DNMTs) expression are known to modulate macrophage differentiation and polarization. A study carried out with obese animals showed an increase in DNMT expression in M1 macrophages and reduction in the M2 macrophages polarized profile, suggesting that DNMT3B functions in the polarization of these cells, mainly through increased PPAR-γ promoter region methylation, a transcription factor important for the polarization of M2 macrophages [11]. Another study also showed that increased expression of DNMT1 in M1 macrophages was related to the induction of methylation in the SOCS1 promoter region, and consequent increase in IL-6 and TNF-α expression by RAW264.7 macrophages after stimulation with LPS [12].

Since epigenetic changes drive cell polarization, manipulation of epigenetics to treat different diseases especially cancer, has now gained increased interest. In this study, we aimed to evaluate the effects of these epidrugs on monocyte and macrophage phenotypes and functions and explore epigenetic manipulation of cell function as a therapeutic strategy. Here, we demonstrate the effects of the DNMT inhibitor (DNMTi), decitabine, which has recently been approved by the FDA for the treatment of myelodysplastic syndrome (MDS) [13,14]. The immunomodulatory effect of this drug was already demonstrated in dendritic cells derived from peripheral blood monocytes, in which the treatment increased costimulatory molecule expression such as CD40 and CD86, after the LPS challenge [15]. In addition, decitabine administration in natural killer (NK) cells improved control tumor growth by increasing IFN-γ release [16]. These data support the potential immunomodulatory role of DNMTi in immune cells and reinforce the importance of studying its effects on monocyte and macrophage function.

Here, we show that decitabine treatment increases phagocytosis and IL-8 production by monocytes, in response to mycobacterial infection. However, its administration reduces pathogen killing. Based on these data, we also determined the effect of drug administration on the macrophage phenotype and observed an increase in M2 markers, indicating a possible induction of alternative polarization, despite no differences in inflammatory cytokine release. Finally, in an in vitro granuloma-like model with *Mycobacterium tuberculosis* (Mtb), corroborating the induction of M2 macrophage phenotypes, treatment with decitabine reduced the proinflammatory cytokines, with equal bacterial burden control, suggesting a potential role of decitabine in regulating the inflammatory response in infectious diseases.

## 2. Materials and Methods

### 2.1. Ethical Aspects

This research was approved by the Ethics Committee of Faculdade de Ciências Farmacêuticas de Ribeirão Preto–FCFRP-USP (CEP/FCFRP nº. 421–CAAE nº 59466716.1.0000.5403). All participants provided written informed consent for participation in the study.

### 2.2. Isolation of Human Mononuclear Cells

Peripheral blood samples from 30 healthy individuals were collected in heparin-containing Vacutainer tubes (BD Bioscience, San Diego, CA, USA) and centrifuged to obtain mononuclear cells according to a standard protocol. Following centrifugation at 400× *g* for 10 min, the cellular portion was separated, diluted in phosphate buffered saline solution (PBS), and applied to a density gradient Ficoll (GE Healthcare, Uppsala, Sweden) and then centrifuged for 30 min at 600× *g* at 25 °C. The fraction of mononuclear cells (PBMCs) was collected from the gradient interface and washed twice with PBS. Thereafter, the monocytes were purified from PBMCs by positive immunomagnetic selection using a kit (Miltenyi Biotec, Auburn, CA, USA). The cells from the mononuclear fraction were added to beads complexed with anti-CD14 monoclonal antibodies and the solution was applied to a magnetic separation column. The adherent cells, which correspond to monocytes (fraction CD14^+^), were obtained through elution. The viability and the number of cells were determined using Trypan Blue (Gibco, Grand Island, NE, USA) and a Neubauer chamber. The number of individuals per experiment is described in the figure legends.

### 2.3. Treatment of Cells with DNMTi

Cell suspensions were treated with 5-AZA-2-deoxycytidin (decitabine or deci) (Sigma-Aldrich, St. Louis, MI, USA) reconstituted in DMSO (0.2%) (Sigma-Aldrich, at concentrations of 5 μM, 1 μM, and 0.25 μM for a period of 24 h before analysis. The concentration of 5 μM was determined according to plasma levels achieved in patients who are treated with decitabine [17,18] and the dose used in other studies evaluating the immunomodulatory effects of these compounds [15,16].

### 2.4. Bacterial Growth and Infection

The H37Rv strain of *M. tuberculosis* (American Type Culture Collection, Rockville, MD, USA) was grown in 7H9 medium for 11 days. The culture was washed by centrifugation, and the pellet resuspended in sterile PBS. The bacterial suspension density was adjusted according to nephelometric McFarland scale to 1 × 10^7^ bac/mL. Bacteria were centrifuged and resuspended in an equal volume of culture medium. The in vitro infection of monocytes/mononuclear cells was performed using multiplicity of infection (MOI) 5 (5 bacteria per cell) at 37 °C and 5% CO_2_, and the time of infection was established according to the experiment.

### 2.5. Cellular Viability Analysis

To assess the viability of monocytes, cells were cultured in the presence of different concentrations of decitabine and controls. Subsequently, resazurin (1 mg/mL) was added to cells treated for 24 h with the epigenetic compound and incubated at 37 °C and 5% CO_2_, for viability determination. After 24 h of metabolization, the relative fluorescence units (RFUs) were obtained using a spectrofluorometer (Paradigm SpectraMax, Molecular Devices, Sunnyvale, CA, USA) with excitation at 560 nm and emission at 590 nm. In granuloma-like assays, cell viability was determined by flow cytometry using a Live/DeadTM Fixable Violet Dead cells staining kit (Thermo Fisher Scientific, Waltham, MA, USA). For this, cells were incubated with a dye supplied with a kit used for labeling dead cells. The percentage of labeled cells was determined by flow cytometry FACS Canto II (BD Biosciences, San Diego, CA, USA) and the analysis was performed using FlowJo software v7.6.5. Dot plots are shown in Supplementary Figure S1.

### 2.6. Phagocytic Activity

Isolated monocytes were adjusted to 1 × 10^5^ cells/well, treated with decitabine at different concentrations for 24 h, and infected with Mtb, as described previously. After 2 h of infection, which is the period determined for phagocytosis evaluation, cells were washed with PBS at room temperature, in order to remove non-internalized bacteria. Sequentially, saponin (0.05%) (Sigma-Aldrich) was added to promote lysis and externalization of phagocytized Mtb into the supernatant. The number of internalized bacteria was determined indirectly through resazurin metabolism (50 µg/mL, Sigma-Aldrich) after 24 h of incubation at 5% CO_2_, 37 °C. Phagocytosis was quantified by detecting the fluorescence intensity of the metabolite resofurin in a spectrofluorometer (Paradigm SpectraMax, Molecular Devices, Sunnyvale, CA, USA) with excitation at 560 nm and emission at 590 nm [19]. Data were expressed in RFU and phagocytic index, which corresponds to the percentage of bacterial metabolization in treated groups, compared to untreated monocytes, which was considered as 100% phagocytosis.

### 2.7. Killing Activity

To determine the effect of decitabine treatment on the ability of monocytes to eliminate pathogens, isolated monocytes were adjusted to 2 × 10^5^ per well and incubated at 37 °C, 5% CO_2_. On the following day, the cells were treated with 5 μM decitabine or vehicle, infected with bacterial suspension adjusted to MOI 5 and then incubated again at 37 °C, 5% CO_2_ for 24 h, the period standardized for evaluation of the activity of the microbicide, as in [19]. After this period, 50 μL of saponin (0.2%) was added to the plate for cell lysis and exposure of the internalized bacteria, followed by the addition of 10 μL of resazurin (50 μg/mL). The number of viable bacteria was determined indirectly through resazurin metabolism (50 µg/mL, Sigma-Aldrich) after 24 h of incubation at 5% CO_2_ at 37 °C. The results were based on the detection of fluorescence intensity of the metabolite resofurin in a spectrofluorometer (Paradigm SpectraMax, Molecular Devices, Sunnyvale, CA, USA) with excitation at 560 nm and emission at 590 nm. Data were expressed in RFU and microbicidal index, which corresponds to the percentage of viable bacterial metabolization in treated groups, in relation to untreated monocytes. In some experiments, as granuloma-like models, the recovered bacteria after the killing period were determined by counting the number of colony forming units (CFU) in 7H11 agar plates.

### 2.8. Immunophenotypic Characterization by Flow Cytometry

Cell surface markers were evaluated by flow cytometry, for cellular characterization after treatment with decitabine. The cell suspension obtained after the experimental protocol was adjusted to 1 × 10^6^, centrifuged at 400× *g*, and resuspended in 100 μL PBS containing 2% fetal bovine serum (FBS) and a mixture of antibodies CD206 Alexa Fluor^®^ 488 (clone 15-2) and CD86 PE (clone IT2.2), purchased from BioLegend (BioLegend Inc., San Diego, CA, USA). For labeling, cells were incubated for 25 min at 4 °C in the dark and then washed with 2 mL of 2% PBS at 400× *g* for 10 min at 4 °C. The cell pellet was resuspended in 200 μL PBS with 1% formaldehyde, for labeling, until the time of reading. Expression analyses were performed using FACS Canto II (BD Biosciences, San Diego, CA, USA) and the data analysis performed using FlowJo software v7.6.5. Dot plots are showed in Supplementary Figure S2.

### 2.9. Quantification of Cytokines and Chemokines.

The detection of soluble immune mediators in culture supernatants was performed through a multiplex platform using the Magnetic Luminex Assay kit (R&D Systems, Minneapolis, MN, USA), according to the manufacturer’s methodology or using conventional ELISA (R&D systems, Minneapolis, MN, USA). The cytokines and chemokines quantified were IL-1β, IL-8, IL-10, IFN-γ, and TNF-α. Multiplex assays were carried out using a Luminex Multiplexing Instrument reader (EMD Millipore, Luminex Corporation, Austin, TX, USA), and data analysis was performed using MilliplexAnalyst^®^ software. Conventional ELISA was performed by the addition of the TMD substrate, followed by the addition of 2 N H_2_SO_4_ as the stop solution_._ The absorbance of the samples and the linear standard curve were obtained by spectrophotometric measurement at 450 nm (μQuant, Biotek Instruments Inc., Winusky, VT, USA) and the concentration of analytes determined from the standard curve.

### 2.10. Differentiation and Polarization of Macrophages

Macrophages were obtained through monocyte differentiation in vitro. Monocytes were adjusted to 1 × 10^6^ cells per well and distributed in 24-well plates in DMEM medium supplemented with 10% FBS and recombinant human cytokine GM-CSF (50 ng/mL), according to the protocol of differentiation and polarization described by Kittan 2013 [20]. For evaluation of the treatment effect, 5 μM of decitabine or 0.2% of vehicle (DMSO) was added during the differentiation period. The cells were then incubated at 37 °C and 5% CO_2_ for six days. On day 3, fresh DMEM supplemented with GM-CSF and treatments (decitabine or DMSO) was added to the culture. After 6 days of incubation, cells were washed with PBS, and fresh media supplemented with the polarizing cytokines were added to induce M1, by addition of IFN-γ (100 ng/mL) and for M2, IL-4, and IL-13 at a concentration of 10 ng/mL. After 24 h of stimulation with the polarizing cytokines, the cells were used to evaluate the expression of surface markers by flow cytometry and gene expression analysis. Functional assays of the differentiated macrophages through stimulation with Mtb were also performed. For this, macrophages were infected with Mtb (MOI 5) and incubated at 37 °C and 5% CO_2_ for a further 24 h. The next day, the supernatant was collected for quantification of cytokines, and the ability to control the infection was determined by counting the bacilli (CFUs) after the period of infection.

### 2.11. Induction of Granuloma-Like Structures

Granuloma-like cell aggregates were induced by a previously described methodology [21,22,23]. To this end, isolated PBMCs were adjusted at 1 × 10^6^ cells per well in RPMI 1640 medium supplemented with 10% FBS and distributed in 48-well plates. The plates were precoated with agarose (230 μL/well) to prevent cell adhesion and promote the formation of cell aggregates. The cells were then treated with 5 μM decitabine or DMSO (0.2%) and incubated at 37 °C and 5% CO_2_ for 24 h. After this period, 1 × 10^4^ bacilli were added to each well, and then the infected mononuclear cells were incubated at 37 °C and 5% CO_2_ for 7 days. The hypomethylating agent treatment was boosted after 3 days of infection. The formation of the cell aggregate structures was evaluated using an inverted microscope and photographed at the end of the 7 days of infection (data not shown). To evaluate the effect of treatment on cellular function after 7 days of infection, the supernatant was collected for quantification of the cytokines IFN-γ, IL-10, TNF-α, and IL-1β by the enzyme immunoassay. Cells were recovered for evaluation of cell viability by flow cytometry. Finally, the ability to control Mtb infection was determined by counting viable CFU in each condition, after cell lysis by saponin (0.05%).

### 2.12. Gene Expression Analysis

RNA extraction was initially performed using the PureLink™ RNA minikit kit (Ambion, Life Technologies™, Carlsbad, CA, USA) for evaluation of gene expression, according to the manufacturer’s instructions. After elution, the recovered RNA was quantified using a NanoDrop^®^2000 spectrophotometer, with a 260/280 ratio close to 1.80. The RNA samples were converted using PCR, into complementary DNA (cDNA), using the cDNA High Capacity Archive kit (Applied Biosystems, Foster City, CA, USA). Finally, after cDNA synthesis, target gene expression was determined by real-time PCR. The reaction was performed by adding 10 μL of GoTaq^®^ Probe qPCR Master Mix (Promega, Madison, WI, USA), 1 μL primer, and 9 μL cDNA (50 ng), and nuclease-free water, to a final volume of 20 μL, and performed on StepOnePlus™ equipment (Applied Biosystems, Foster City, CA, USA). The normalization of the Ct results (cycle threshold) of the target genes, *ALOX15* and *CXCL10*, was performed in reference to the constitutive β-actin gene (*ACTB*). Data were analyzed by relative expression to untreated groups, using 2^−ΔΔCt^ for expression calculation.

### 2.13. Statistical Analysis

Statistical analyses were performed using GraphPad Prism 8 (GraphPad Software Inc., San Diego, CA, USA). Statistical tests were chosen according to data distribution analysis, determined through the Kolmogorov–Smirnov normality test using Dallal–Wilkinson–Lillie for the *p*-value. Paired *t*-test was used for the parametric samples, for the comparison of two groups, and ANOVA for repeated measures, followed by a Tukey test, was used for comparison between the groups in analysis with more than two groups. For non-parametric data, we used the Friedman test, followed by Dunn’s test for paired multiple comparisons, and the Wilcoxon test, for paired analyses between two experimental groups. Data are presented as mean ± standard deviation, considering *p* values less than 0.05 (two-tailed) to be statistically significant.

## 3. Results

### 3.1. Treatment with a Hypomethylating Agent Modulates Monocyte Functions in Response to Infectious Stimuli

**Figure 1:**
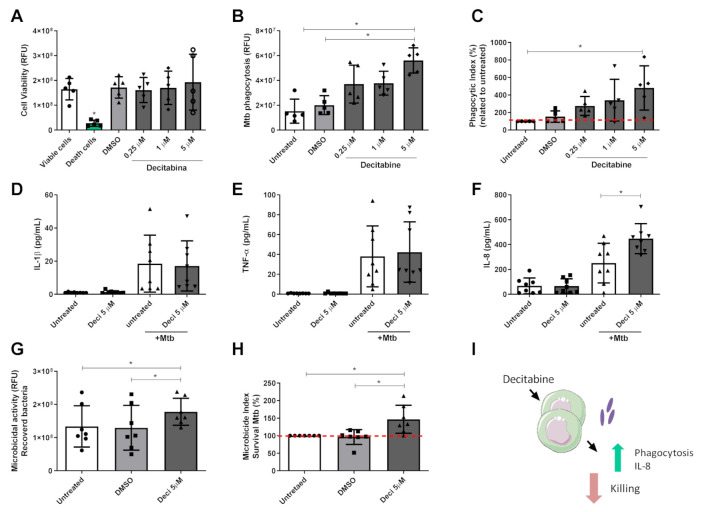
Decitabine treatment modulated the monocyte response to mycobacterial infection. Monocytes were isolated from healthy individuals and pretreated with decitabine (0.25–5 µM), DMSO, or untreated for 24 h. After this period, cell viability was assessed by detecting resazurin metabolism (**A**). In addition, cells were infected with Mtb (MOI 5) for different periods. For phagocytosis analysis, cells were infected for 2 h, followed by washes with PBS, saponin lyses, and exposure to internalized bacteria. The number of phagocytized bacteria was determined indirectly by detecting resazurin metabolism, expressed in (**B**) and phagocytic index, normalized in relation to untreated cells, considered as 100% (**C**). Immune mediator release was determined after 24 h of pretreated monocyte infection with Mtb and quantified using a Magnetic Luminex Assay kit, as demonstrated in (**D**) IL-1β, (**E**) TNF-α, and (**F**) IL-8. (**G**,**H**) Microbicide activity was evaluated after 24 h of infection and the number of bacteria recovered from cells was quantified indirectly by detecting resazurin metabolization, expressed in (**G**), and the killing index was determined by normalization of RFU quantitation, in relation to untreated cells, considered as 100% (**H**). Data are expressed as mean ± standard deviation and analyzed by paired ANOVA, followed by a Tukey’s test. Phagocytosis and killing index were analyzed by a Friedman test (non-parametric), followed by a Dunn’s test. All the analyses consider * *p* < 0.05 as statistically significant (**I**) A schematic summarizing the effects of decitabine on monocyte function during Mtb infection.

Before evaluating the modulation exerted by decitabine on the functional aspects of these cells, we determined the effect of treatment on cell viability. For this, monocytes were plated onto culture plates and treated with different concentrations of decitabine for 24 h. The concentrations used and the established treatment period were based on other studies, which evaluated the modulation by decitabine on the function of other immune cells, such as NK and dendritic cells [15,16]. Cell viability was determined after incubation, by evaluating the cellular metabolism of resazurin. Cell metabolism after the addition of lysis solution (saponin 0.05%) was used as a positive control for cell death, and cells that did not receive any type of treatment were considered as viability controls (Figure 1A).

Since no cellular toxicity was observed in our study model, we next evaluated the effect of the treatment on the functional activities of the cells. Phagocytic capacity of monocytes, an important function in the control of infectious processes, was tested by treating the monocytes for 24 h with different concentrations of the hypomethylating compound or DMSO. As demonstrated, treatment with decitabine at the highest concentration (5 μM) resulted in greater RFU detection when compared to the untreated control group or with cells receiving only the vehicle DMSO, indicating the presence of a higher number of bacteria in these cells after treatment, and, consequently, an increase in phagocytosis (Figure 1B).

Considering the individual variation in phagocytosis rates, we normalized the data for each individual in relation to their untreated cells, considering 100% phagocytosis. Thus, in Figure 1C, as determined by RFU, treatment with 5 μM decitabine promoted an increase in the phagocytic index in relation to the untreated group, with an approximately 4-fold increase in the ability of these cells to recognize and internalize the bacillus.

Given the positive modulation of decitabine treatment on phagocytic activity, we next determined the effect of this treatment on the release of soluble mediators by monocytes, which represents a fundamental function of these cells for the development and orchestration of an appropriate immune response. After 24 h of Mtb challenge, the supernatant was used to quantify the immune mediators produced in response to the infection. No change was observed in the production of IL-1β and TNF-α (Figure 1D,E, respectively). However, the treatment with 5 μM decitabine promoted an increase in IL-8 cytokine production after stimulation, when compared with untreated infected control (Figure 1F). The quantification of IL-10 was under the detection limit of the applied test (data not shown).

In addition, killing activity was also determined after decitabine treatment. For this, saponin was added to the wells for cell lysis 24 h post infection, followed by resazurin, to estimate the number of bacteria remaining alive after the killing period. A higher fluorescence detection in monocytes treated with decitabine was observed, suggesting a higher number of viable bacteria in this group after the 24 h infection period (Figure 1G). To determine the microbicide index, the percentage of viable bacteria in each individual was analyzed and compared to bacteria recovered from the untreated cells, leading to an observation that the monocytes treated with the hypomethylating compound maintained the reduction present in the infection control capacity, with an elevated percentage of viable bacteria recovered (Figure 1H). Thus, we proposed that, despite increasing the internalization of bacteria and IL-8 production, the treatment impaired the ability of monocytes to control Mtb infection, as summarized in Figure 1I.

### 3.2. Decitabine Potentializes M2 Marker Expression in Macrophages and Leads to Impaired Infection Control

Based on the data regarding monocytes, and previous knowledge regarding the importance of epigenetic changes for the differentiation and polarization of macrophages, our next objective was to determine whether decitabine treatment influences macrophage phenotype and thereby alters the pattern of immune response against Mtb infection. To this end, purified monocytes were cultured for 6 days in a medium supplemented with GM-CSF, in the presence of decitabine or DMSO (vehicle) for differentiation into macrophages. The differentiated macrophages were then stimulated with polarizing cytokines for the M1 profiles, by the addition of IFN-γ and M2, with IL-4 and IL-13 and cells that received only a medium and were maintained in the condition M0 (resting).

**Figure 2:**
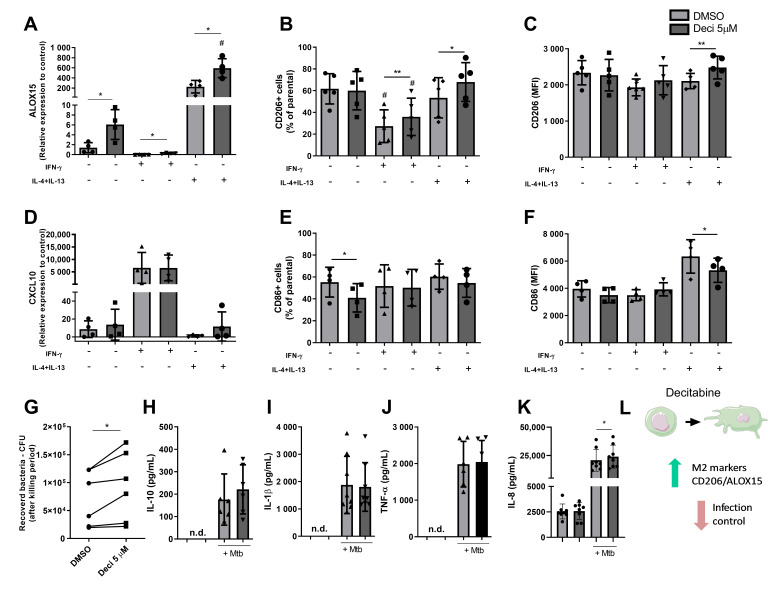
Decitabine modulated macrophage function by potentializing the M2 like profile. Monocytes from healthy subjects were isolated from peripheral blood and cultured for 6 days in the RPMI 1640 medium supplemented with 10% FBS and 50 ng/mL granulocyte-macrophage colony-stimulating factor (GM-CSF), and 5 μM decitabine or DMSO (0.2%) added for macrophage differentiation. On the 6th day, differentiated macrophages were stimulated with cytokines for macrophage polarization or infected with Mtb for functional analysis. The M1 profile was defined by the addition of IFN-γ (100 ng/mL) and M2, by IL-4 and IL-13 (50 ng/mL each). After 24 h in the presence of polarizing cytokines, the macrophage profile was determined by gene expression of *ALOX15* (**A**) and *CXCL10* (**D**). The expression was normalized using Ct of the endogenous gene (*ACTB*) and the relative expression calculated using 2^−ΔΔCt^ in relation to resting macrophages differentiated with DMSO. In addition, surface markers CD206 ((**B**) frequency, and (**C**) Mean Fluorescence Intensity) and CD86 ((**E**), frequency and (**F**), Mean Fluorescent Intensity—MFI) (**D**,**F**) were analyzed by flow cytometry. For functional analysis, after the differentiation period in the presence of decitabine or DMSO, macrophages were infected with Mtb and 24 h post-infection, cells were lysed to bacterial load determination through colony forming units (CFU) count, represented in (**G**), and the supernatant was collected for IL-10 (**H**), IL-1β (**I**), TNF-α (**J**), and IL-8 (**K**) quantitation by ELISA. Data were analyzed by paired tests, according to the normality distribution and number of groups per experiment. Gene expression and surface markers were analyzed through the ANOVA test for repeated measurements, followed by a Tukey test to evaluate the effect of stimulation with polarizing conditions, with # *p* < 0.05 M0 vs. M1 and M2 vs. M1. In addition, the effect of decitabine treatment in each condition was evaluated by the paired *t*-test with * *p* < 0.05 and ** *p* < 0.01 in relation to DMSO. CFU analysis was performed by the paired *t*-test, and cytokine release was analyzed by the non-parametric Wilcoxon test comparing decitabine to DMSO in infected conditions (*). All analyses considered *p* ≤ 0.05 as statistically significant. (**L**) Figure summarizing the effects of decitabine on macrophage phenotypes of polarization and their functions during Mtb infection. The symbols within the bars of the graphs represent each evaluated samples.

Macrophage phenotypes were assessed using surface markers through flow cytometry and by expression of genes associated with the different profiles. The treatment increased gene expression of *ALOX15*, an M2-associated gene, in all polarization conditions, suggesting a possible effect of decitabine treatment in modulating macrophage polarization to an alternative profile (Figure 2A). Furthermore, when macrophages were differentiated in the presence of decitabine, we observed an increase in CD206^+^ cells, compared to those that received only the vehicle under the same polarizing conditions for M1 and M2 profiles. The same modulation was demonstrated in the mean expression of this receptor per cell, represented by mean fluorescence intensity (MFI) (Figure 2B,C, respectively), corroborating a possible modulation of decitabine in macrophages phenotypes. As expected, there was a reduction in the frequency of CD206^+^ cells in M1 polarized condition when compared to the M2 group and resting macrophages, in the absence of epigenetic treatment, confirming our polarization protocol.

In contrast, decitabine did not influence the expression of *CXCL10*, an M1-associated marker, which presented an increased expression in macrophages polarized with IFN-γ, independent of the treatment with the epigenetic modulator (Figure 2D). In addition, we demonstrated a reduction in CD86 expression, mainly in macrophages differentiated in the presence of decitabine, in resting macrophages, and in those polarized to the M2 profile (Figure 2E,F).

Subpopulations of macrophages have different roles in the immune response, producing different patterns of cytokines, dictating the infection outcome. To determine the impact of decitabine on the killing and cytokine production by macrophages, these cells were infected with Mtb and 24 h post infection, CFU, and immune mediator release were assessed. Thus, corroborating the effect of potentiating the polarization to the M2 profile, we observed a greater amount of bacteria recovered after 24 h of infection from macrophages, differentiated in the presence of decitabine, suggesting a reduction in the microbicidal capacity of these cells, as demonstrated by the monocyte experiments (Figure 2G).

In addition to microbicidal activity, the production of cytokines by macrophages after Mtb infection was also determined, as shown in Figure 2H–K. However, unexpectedly, despite increased expression of the markers for the M2 profile, differentiated macrophages in the presence of decitabine did not demonstrate an anti-inflammatory profile of cytokine production, showing very similar levels of IL-10 between DMSO and decitabine-differentiated macrophages. The same was observed for proinflammatory mediators, IL-1β and TNF-α. A slight increase was observed in the IL-8 production by macrophages that were differentiated with decitabine, when compared with DMSO, in infected conditions, corroborating the results already observed in monocytes.

### 3.3. Hypomethylating Agent Reduces Inflammation in a Granuloma-Like Model

It has been well described that during Mtb and other infections, in addition to the modulation of the function of monocytes and macrophages, the interaction of these cells with T lymphocytes can direct the host immune response to ensure disease control. In tuberculosis infection, granuloma formation is an important mechanism of bacterial burden contention. These structures are driven by macrophages, and consequently rely on macrophage polarizations [24,25].

Therefore, we proposed to evaluate the modulation performed in coculture assays of PBMCs, mimicking in vitro granuloma structures. Thus, mononuclear cells were cultured on agarose-coated plates to reduce cell adhesion, favoring the formation of cell aggregates after Mtb infection. In our model, after cell isolation, they were distributed in 48-well plates coated with agarose, treated with DMSO or 5 μM of decitabine, and incubated for 24 h. The cells were then infected with Mtb (MOI 0.01) and the modulatory properties of the hypomethylating agent on cell function evaluated after 7 days, through bacterial burden control and cytokine release measurement.

**Figure 3:**
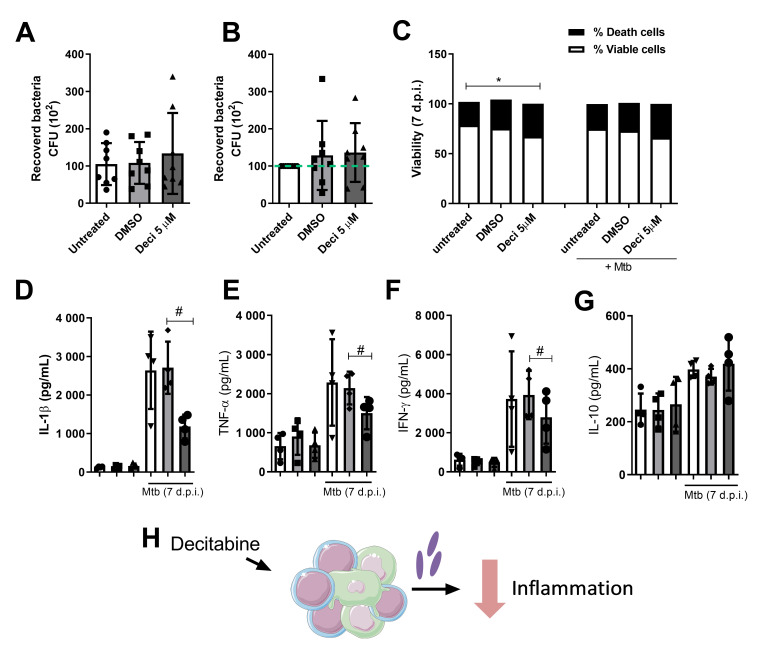
Decitabine reduces inflammation during Mtb infection in granuloma-like models. Peripheral blood mononuclear cells (PBMCs) were isolated and distributed (1 × 10^6^ cells/well) into 48-well plates coated with an agarose layer (230 μL/well). Cells were incubated in RPMI medium supplemented with human serum (10%) for 24 h in the presence of 5 μM decitabine, DMSO, or untreated. The next day, cells were infected with Mtb H37Rv (1 × 10^4^ bacteria/well) and incubated at 37 °C and 5% CO_2_ for 7 days. After the infection period, 0.2% saponin was added to promote cell lysis and recovery of bacteria. The bacterial suspension was diluted in 1X PBS and distributed in 7H11 plates for CFU count after 21 days of incubation. Data are represented as absolute count (**A**) and index of recovered bacteria normalized for each individual, considering untreated cells as 100% (**B**). (**C**) Viability assessment was performed using a Live/DeadTM Fixable Violet Dead cells staining kit (Thermo Fisher Scientific) and detected using a FACS Canto II flow cytometer (BD Biosciences, San Diego, CA, USA) (*n* = 3). The production of IL-1β (**D**), TNF-α (**E**), IFN-γ (**F**), and IL-10 (**G**) were quantified in culture supernatants after 7 days of culture by ELISA. All data were analyzed by ANOVA for repeated samples, followed by Tukey’s post-hoc test, considering * *p* < 0.05 For cytokine analysis, ANOVA for repeated samples, followed by Tukey’s post-hoc test were performed comparing decitabine to controls in infected conditions, considering # *p* < 0.05. (**H**) Figure summarizing the immunomodulation exerted by the hypomethylating agents during coculture of monocytes/macrophages and lymphocytes. The symbols within the bars of the graphs represent evaluated samples.

The number of viable bacilli recovered after 7 days of infection was determined by CFU count and the microbicidal activity modulation by decitabine treatment. As shown in Figure 3, the treatment did not influence the ability of cells to control infection, given the similarity between the mean CFU in the different groups. Due to the large individual variation in bacillus recovery, we calculated the percentage of microbicidal activity, considering the CFU count of the untreated cells as 100%. However, no difference was observed in Mtb killing.

In spite of equal microbicidal activity, it was observed that decitabine modulated the release of inflammatory cytokines, such as IL-1β, TNF-α, and IFN-γ, which showed a significant reduction after 7 days of infection when compared to the DMSO group (Figure 3E–G). This effect was not observed in the amount of anti-inflammatory cytokines released, since treatment did not influence IL-10 production (Figure 3H). However, the ratio between anti- and proinflammatory mediators was higher in treated cocultures, suggesting an anti-inflammatory effect in this case.

This decrease in proinflammatory cytokines produced in response to infection could be the result of cellular toxicity caused by the treatment. Thus, to demonstrate that such effects are a result of immunomodulation, cell viability was assessed by probe labeling by flow cytometry. Cells treated with a hypomethylating agent showed a reduction in viability, with a significant increase in the percentage of dead cells when compared to the non-infected control group. In addition, we demonstrated that infection did not induce changes in cell viability and treatment with epigenetic compounds did not promote cellular mortality (Figure 3C,D). Thus, the modulation of the immune response exerted by decitabine treatment after infection, which was observed in the production of cytokines, was not due to the reduction in viable cells, but rather, corresponded to the effects of the treatment on this cellular function. The effects observed in the coculture model corroborated our findings in polarization of macrophages to M2 profiles, with induction regulatory profiles of the immune response, as summarized in Figure 3I.

## 4. Discussion

The interest in seeking new treatment alternatives for diseases based on epigenetic changes is increasing and, therefore, it is essential to understand the effects of these drugs, not only in target cells, but also in particular, on the phenotype and function of immune cells. The epigenetic alterations and modulation of immune cell function and the modulation exerted by epigenetic treatments have been studied by other research groups. In a recently published review, Lindblad et al. collated studies demonstrating the regulation of treatment with hypomethylating agents on immune cells [26]. However, the function of human monocytes was not discussed to a great length. Thus, knowing the importance of monocytes and macrophages in innate defense mechanisms and in directing the effector functions of T cells, aiding infectious control, tumor response, or tissue remodeling and the role of epigenetic alterations in the regulation of the function and differentiation of these cells, we sought to understand the modulation exerted by in vitro administration of a hypomethylating agent, decitabine, on the function of monocytes and the impact of this treatment on the differentiation and polarization into different profiles of macrophages.

Initially, we demonstrated that treatment with decitabine significantly modulated the phagocytic function of monocytes, increasing the ability of these cells to internalize Mtb. Our data corroborate the increase in phagocytosis observed after treatment with the hypomethylating compound, 5-AZA, which has already been demonstrated in J774 murine macrophages infected with *Paracoccidioides brasiliensis*, after 2 and 6 h of infection [27]. In addition, it is also known that the internalization of microorganisms by monocytes depends on recognition through receptors such as Toll-like receptors (TLRs) [28]. Another study described the association between methylation and TLR expression and showed that the reduction in TLR2 expression during *Porphyromonas gingivalis* infection in deregulated gingival epithelial cells was caused by hypermethylation of the promoter of this gene. In addition, the authors demonstrated that the treatment of these cells with DNMT inhibitors (1 μM decitabine) has been shown to recover TLR2 expression in oral mucosal cells and also appear to be important in inducing an inflammatory response during infection [29]. Despite some evidence in literature, the mechanisms that lead to an increase in phagocytosis observed in our data were not completely understood and more investigations are still needed to elucidate how decitabine promote these alterations in the monocytes function.

In addition, we also observed an increase in IL-8 production after Mtb infection in monocytes pretreated with decitabine. The regulation of the expression and release of this cytokine is directly associated with changes in DNA methylation pattern, which has already been demonstrated in colorectal cancer models and cases of inflammation in the oral mucosa [30,31]. Thus, the role of the agent in inhibiting DNA methylation, and the important regulation that this mechanism exerts in the production of this mediator, can explain the observed increase in cells treated with decitabine, in our model. Functionally, IL-8 production by monocytes and macrophages is induced in the early stages of Mtb infection or other infections and it is responsible for the recruitment of other inflammatory cells, mainly neutrophils [32,33]. Additionally, a recent study has shown that IL-8 interacts with Mtb and that this association directly leads to increased phagocytosis and death of bacillus by inflammatory cells [34], providing support for the increased phagocytosis observed in our data.

On the other hand, production of this cytokine has already been clearly associated with tumor progression. Studies have shown that IL-8, along with IL-6, TGF-β, and metalloproteinases, correspond to the major mediators produced by tumor-associated macrophages (TAMs) and is associated with angiogenesis, metastasis, and, consequently, tumor progression [35,36]. Together with this evidence, despite the increase in phagocytosis, the treatment induced a reduction in the microbicidal activity of monocytes, with a higher bacterial load recovered after 24 h of infection, demonstrating less control of the infectious process. Thus, the modulation exerted until now, highlighting the increased phagocytic activity and lower microbicidal capacity and the modulation of chemokine production, with increased IL-8, presented great similarity with the function performed by macrophages polarized to the M2 profile. Therefore, we hypothesized a potential modulation of M2-like macrophages after treatment with decitabine.

Supporting our hypothesis, the role of epigenetic changes, in especially DNMTs regulation, during macrophage polarization has already been demonstrated [4,37]. A study carried out with obese mice reported an increase in the expression of DNMT enzymes in M1 macrophages and reduction of expression in M2 polarized macrophages, suggesting a DNMT3B role in the polarization of these cells, mainly for inducing the increase in methylation of the PPAR-γ promoter region, a transcription factor important for the polarization of M2 macrophages [11]. In addition, another study demonstrated that treatment ofmacrophages with 5-AZAdC increased ARG1 expression in these cells, and increased the population of F4/80^+^CD206^+^ M2 macrophages and reduced TNF-α production, controlling inflammation response in obesity [38]. Additionally, it was already demonstrated that increased expression of DNMT3B enzyme in M1 macrophages is related to the induction of hyper-methylation in the SOCS1 promoter region, a negative regulator of inflammatory cytokines release, and consequently, it was observed an increase in IL-6 and TNF-α expression by RAW264.7 macrophages after stimulation with LPS [12].

We demonstrated that the presence of decitabine during differentiation potentiated the expression of surface markers characteristic of the M2 profile, such as CD206, and reduced the expression of CD86, molecules with higher expression in M1 macrophages. In addition, ALOX15, a gene related to the M2 profile, was also differentially expressed in the presence of the hypomethylating compound, demonstrating increased expression in resting and after polarization with IL-4 and IL-13. Although it did not prevent the polarization to the inflammatory profile in the presence of IFN-γ, as demonstrated by the similarity in *CXCL10* expression, a greater expression of *ALOX15* in this condition when the macrophages were differentiated with decitabine was observed. It is possible that the treatment with epigenetic regulators could induce the modulation of cytokine receptors expression in our model, like IL-4 and IFN-γ, explaining the polarization effects. Future studies are needed to affirm this hypothesis.

Our data corroborate data from other studies that demonstrated that DNMT inhibitors reduce inflammation by acting on macrophage polarization and are therefore attractive strategies to control infections such as sepsis and inflammatory diseases. In a murine model of acute lung injury induced by LPS, treatment with a combination of DNMT and histone deacetylase (HDAC) inhibitors, azacitidine and tricostatin A (TSA), reduced pulmonary inflammation, with decreased cell recruitment, and consequently the inflammatory infiltrate. The improvement in the clinical score observed was due to alterations in the subpopulations of macrophages, favoring inflammatory response regulation. Thus, the treatment was shown to promote the reduction of proinflammatory mediators such as CXCL10, IL-6, IL-1β, and TNFα, while increasing the amount of IL-10 as detected. Furthermore, it was possible to observe an increase in the frequency of arginase-1 (Arg1) positive cells, an important M2 marker in mice [39].

Another example was demonstrated in a model of acute heart attack, in which treatment with the DNMTi showed improvement in cardiac injury by promoting the reduction in inflammation and iNOS expression and, consequently, a decrease in the production of nitric oxide. This reduction was accompanied by an increase in CD206 positive cells in the cardiac infiltrate and M2 profile cytokines, such as IL-4. Therefore, the authors suggested that 5-AZA aids in the control of cardiac injury by potentiating the polarization of M2 macrophages [40].

Despite M2 markers, the infection of these macrophages with Mtb demonstrated an atypical profile of response, with poor control of infection and similar release of immune mediators, such as IL-10, IL-1β, and TNF-α. The diverse range of M2 responses has already been discussed, and some evidence indicates that Mtb infection could induce atypical M2 macrophages, which exhibit M1 and M2 associated gene transcription patterns [8,41], as observed in our model. These macrophages express M2 surface markers, such as CD206, Ym1, and CD163, and produce several inflammatory mediators, including IL-12, IL-10, TNF-α, and IL-6 [42,43]. In addition, the role of M2 macrophages in tuberculosis is already controversial, and it has already been reported that M2 macrophages, in inflammatory conditions such as in the lung microenvironment during Mtb infection with INF-γ, could increase their bactericidal activity accompanied by less tissue damage, promoting resistance to tuberculosis disease [44].

Based on this, we aimed to evaluate the regulation of the hypomethylating agent during monocyte/macrophage and lymphocyte coculture, mimicking the formation of granuloma-like structures. In this model, we showed a reduction in the release of inflammatory mediators. Levels of IFN-γ, TNF-α, and IL-1β were significantly reduced after 7 days of infection in the cultures treated with decitabine, but the quantification of IL-10 remained unchanged, suggesting an increase in anti-inflammatory ratio and control of inflammation. This decrease in inflammation was due to functional modulation of these cells, since cellular toxicity of the compound was absent, with maintenance of similar viability between the groups after infection. Moreover, although more bacilli were recovered from the treated cocultures, we did not observe significant differences between groups, suggesting that the treatment promoted similar infection control, while regulating inflammation, which may have led to less tissue damage.

The regulation of inflammatory responses has already been demonstrated in decitabine treatment. During experimental autoimmune encephalomyelitis, ex vivo experiments demonstrated that treatment with decitabine reduced the T cell infiltrate in the central nervous system and the activation of resident macrophages, with reduction in CD45 and MHC class II expression. Furthermore, it was shown that the treated animals presented lower gene expression of cell damage mediators, such as *TNF-α*, *IL-β*, and *iNOS*, concomitantly with a decrease in inflammatory cytokines and chemokines IL-6, IL-12, and CXCL10, which are associated with the recruitment of inflammatory cells. Positive effects of decitabine treatment on cardiac transplant rejection were also observed, with increased survival of the treated group. This clinical improvement is associated with modulation of the T cell function, by reducing the proliferation and differentiation of cells into the Th1 and Th17 profiles and in the production of IL-17, IFN-γ, and TNF-α by spleen cells from treated animals [45]. In addition, another group also demonstrated a modulation in regulatory T cell subpopulation, as observed in a model of experimental autoimmune neuritis, mimicking Guillain–Barré syndrome, in which the treatment with decitabine demonstrated a reduction in the clinical score by increasing the frequency of FOXP3^+^ cells in the thymus, reducing Th1 and Th17 cells in treated animals and inducing a reduction in the production of proinflammatory cytokines by spleen cells stimulated in vitro [46].

Thus, our results corroborate some data from other studies, but provide evidence of immunomodulatory effects of hypomethylating agents in human macrophages, reaffirming a potential regulatory role of decitabine in monocyte and macrophage function. Here, we demonstrated that decitabine could potentialize M2 polarization and might be a therapeutic strategy in controlling exacerbated immune response during inflammatory diseases, opening new avenues on studies of drug repositioning or incrementing combined immunotherapy strategies.

## Data Availability

The data presented in this study are available on request from the corresponding author. The data are not publicly available due to ethical reasons.

## Supplementary Materials

The supplementary material is available online: https://www.mdpi.com/article/10.3390/cells10040868/s1, Figure S1: Cytometry representative dot plots from viability analysis; Figure S2: Cytometry representative gate strategy and histogram from macrophages surface markers analysis.
