# Supplementary material for: Decitabine Promotes Modulation in Phenotype and Function of Monocytes and Macrophages That Drive Immune Response Regulation
Source: Cells. 2021 Apr 12;10(4):868. doi: 10.3390/cells10040868 (PMC8069756; doi:10.3390/cells10040868)
Supplement: Supplementary file 1 [file cells-10-00868-s001.zip › Sup_FAZ/Figure captions- FAZ - 01-12-2020.docx]

**Figure 1.** **Decitabine treatment modulated the monocyte response to mycobacterial infection.** Monocytes were isolated from healthy individuals and pretreated with decitabine (0.25-5 µM), DMSO, or untreated for 24 h. After this period, cell viability was assessed by detecting resazurin metabolism **(A).** In addition, cells were infected with Mtb (MOI 5) for different periods. For phagocytosis analysis, cells were infected for 2 h, followed by washes with PBS, saponin lyses, and exposure to internalized bacteria. The number of phagocytized bacteria was determined indirectly by detecting resazurin metabolism, expressed in **(B)** and phagocytic index, normalized in relation to untreated cells, considered as 100% **(C).** Immune mediator release was determined after 24 h of pretreated monocyte infection with Mtb and quantified using a Magnetic Luminex Assay kit, as demonstrated in **(D)** IL-1β, **(E)** TNF-α, and **(F)** IL-8. **(G and H)** Microbicide activity was evaluated after 24 h of infection and the number of bacteria recovered from cells was quantified indirectly by detecting resazurin metabolization, expressed in **(G),** and the killing index was determined by normalization of RFU quantitation, in relation to untreated cells, considered as 100% **(H)**. Data are expressed as mean ± standard deviation and analyzed by paired ANOVA, followed by Tukey’s test. Phagocytosis and killing index were analyzed by Friedman tets (non-parametric), followed by Dunn´s test. All de analysis considers *p*<0.05 as statistically significant **(I)** A schematic summarizing the effects of decitabine on monocyte function during Mtb infection.

**Figure 2. Decitabine modulated macrophage function by potentializing M2 like profile.** Monocytes from healthy subjects were isolated from peripheral blood and cultured for 6 days in RPMI medium supplemented with 10% FBS and 50 ng/mL GM-CSF, and 5 μM decitabine or DMSO (0.2%) added for macrophage differentiation. On the 6^th^ day, differentiated macrophages were stimulated with cytokines for macrophage polarization or infected with Mtb for functional analysis. The M1 profile was defined by the addition of IFN-γ (100 ng/mL) and M2, by IL-4 and IL-13 (50 ng/mL each). After 24 h in the presence of polarizing cytokines, macrophage profile was determined by gene expression of *ALOX15* **(A)** and *CXCL10* **(D)**. The expression was normalized using Ct of the endogenous gene (*ACTB*) and the relative expression calculated using 2^^(-ΔΔCt)^ in relation to resting macrophages differentiated with DMSO. In addition, surface markers CD206 **(B and C)** and CD86 **(D and F)** were analyzed by flow cytometry. For functional analysis, after the differentiation period in the presence of decitabine or DMSO, macrophages were infected with Mtb and 24 h post-infection, cells were lysed to bacterial load determination through CFU count, represented in **(G),** and the supernatant was collected for IL-10 **(H)**, IL-1β **(I)**, TNF-α **(J),** and IL-8 **(K)** quantitation by ELISA. Data were analyzed by paired tests, according to the normality distribution and number of groups per experiment. Gene expression and surface markers were analyzed through ANOVA test for repeated measurements, followed by Tukey test to evaluate the effect of stimulation with polarizing conditions, with *p*<0.05 (#) M0 vs. M1, M2 vs M1. In addition, the effect of decitabine treatment in each condition was evaluated by the paired t-test with *p*<0.05 (*) and *p*<0.01 (**) in relation to DMSO. CFU analysis was performed by the paired t-test, and cytokine release was analyzed by non-parametric Wilcoxon test comparing decitabine to DMSO in infected conditions (*). All analyses considered *p*≤0.05 as statistically significant. **(L)** Figure summarizing the effects of decitabine on macrophage phenotypes of polarization and their functions during Mtb infection.

**Figure 3. Decitabine reduces inflammation during Mtb infection in granuloma-like models.** Peripheral blood mononuclear cells (PBMCs) were isolated and distributed (1x10^6^ cells/well) into 48-well plates coated with an agarose layer (230 μL/well). Cells were incubated in RPMI medium supplemented with human serum (10%) for 24 h in the presence of 5 μM decitabine, DMSO, or untreated. The next day, cells were infected with Mtb H37Rv (1x10^4^ bacteria/well) and incubated at 37 °C and 5% CO_2_ for 7 days. After the infection period, 0.2% saponin was added to promote cell lysis and recovery of bacteria. The bacterial suspension was diluted in 1X PBS and distributed in 7H11 plates for CFU count after 21 days of incubation. Data are represented as absolute count **(A)** and index of recovered bacteria normalized for each individual, considering untreated cells as 100% **(B).** **(C)** Viability assessment was performed using a Live/DeadTM Fixable Violet Dead cells staining kit (Thermo Fisher Scientific, Massachusetts, EUA) and detected using flow cytometer (BD Biosciences, San Diego, USA) (n = 3). The production of IL-1β **(E),** TNF-α **(F)**, IFN-γ **(G),** and IL-10 **(H)** were quantified in culture supernatants after 7 days of culture by ELISA. Data were analyzed by ANOVA for repeated samples, followed by Tukey's post-hoc test, considering *p*<0.05. (#) For cytokine analysis, ANOVA for repeated samples, followed by Tukey's post-hoc test were performed comparing decitabine to controls in infected conditions, considering *p*<0.05 **(I)** Figure summarizing the immunomodulation exerted by the hypomethylating agents during coculture of monocytes/macrophages and lymphocytes.

**Supplementary Figure S1. Cytometry representative dot plots from viability analysis**. Peripheral blood mononuclear cells (PBMCs) were isolated and distributed (1x10^6^ cells/well) into 48-well plates coated with an agarose layer (230 μL/well). Cells were incubated in RPMI medium supplemented with human serum (10%) for 24 h in the presence of 5 μM decitabine, DMSO, or untreated. The next day, cells were infected with Mtb H37Rv (1x10^4^ bacteria/well) and incubated at 37 °C and 5% CO_2_ for 7 days. After this period, viability assessment was performed using a Live/DeadTM Fixable Violet Dead cell staining kit (Thermo Fisher Scientific, Massachusetts, EUA) and detected using flow cytometer (BD Biosciences, San Diego, USA). Dot plots are representative from one of three donors analyzed.

**Supplementary Figure S2. Cytometry representative gate strategy and histogram from macrophages surface markers analysis.** Monocytes from healthy subjects were isolated from peripheral blood and cultured for 6 days in RPMI medium supplemented with 10% FBS and 50 ng/mL GM-CSF, and 5 μM decitabine or DMSO (0.2%) added for macrophage differentiation. On the 6^th^ day, differentiated macrophages were stimulated with cytokines for macrophage polarization or infected with Mtb for functional analysis. The M1 profile was defined by the addition of IFN-γ (100 ng/mL) and M2, by IL-4 and IL-13 (50 ng/mL each). M1 and M2 surface markers expression, CD86 and CD206, were analyzed by flow cytometry. Gate strategy and histograms are representative of different experimental conditions from one donor.
