# Supplementary material for: Decitabine Promotes Modulation in Phenotype and Function of Monocytes and Macrophages That Drive Immune Response Regulation
Source: Cells. 2021 Apr 12;10(4):868. doi: 10.3390/cells10040868 (PMC8069756; doi:10.3390/cells10040868)
Supplement: Supplementary file 1 [file cells-10-00868-s001.zip › Sup_FAZ/Suplemmentary material.docx]

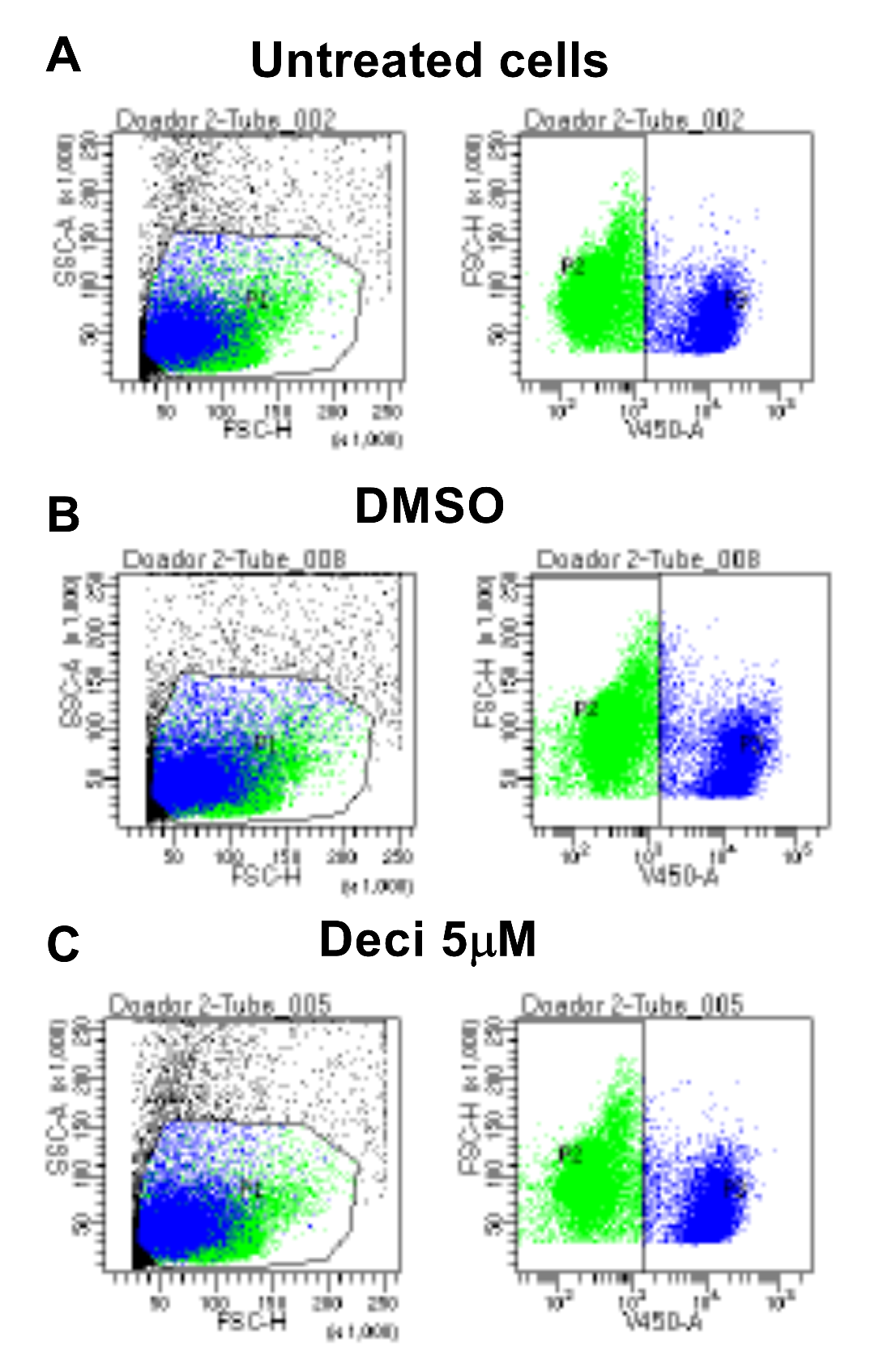


**Supplementary Figure S1. Cytometry representative dot plots from viability analysis**. Peripheral blood mononuclear cells (PBMCs) were isolated and distributed (1x10^6^ cells/well) into 48-well plates coated with an agarose layer (230 μL/well). Cells were incubated in RPMI medium supplemented with human serum (10%) for 24 h in the presence of 5 μM decitabine, DMSO, or untreated. The next day, cells were infected with Mtb H37Rv (1x10^4^ bacteria/well) and incubated at 37 °C and 5% CO_2_ for 7 days. After this period, viability assessment was performed using a Live/DeadTM Fixable Violet Dead cell staining kit (Thermo Fisher Scientific, Massachusetts, EUA) and detected using flow cytometer (BD Biosciences, San Diego, USA). Dot plots are representative from one of three donors analyzed.


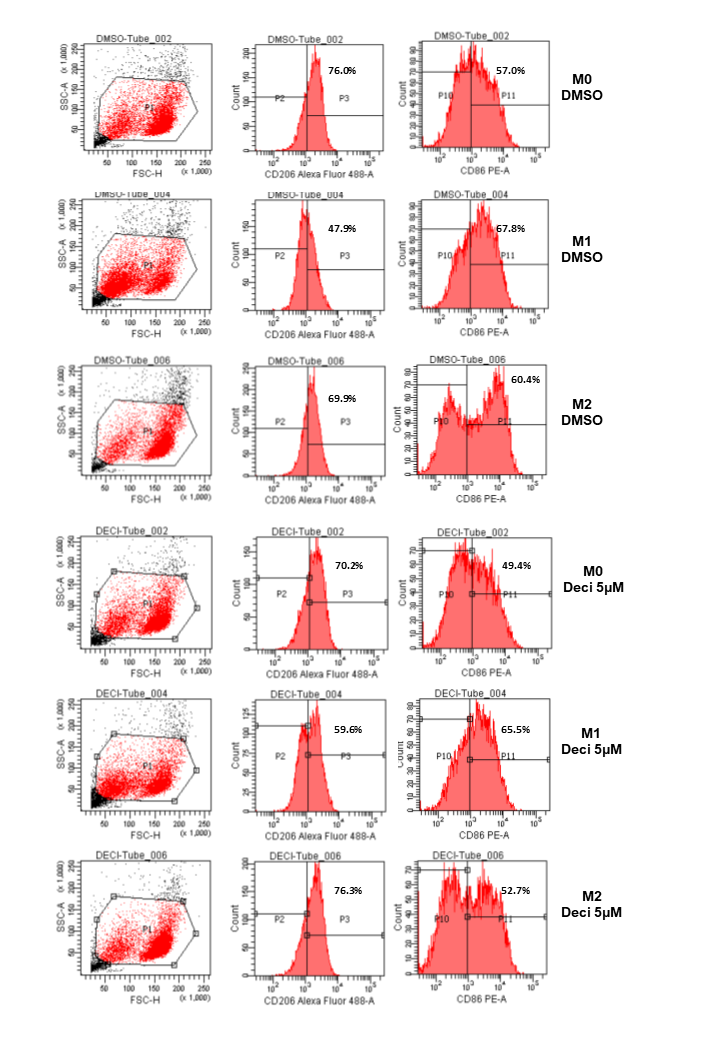


**Supplementary Figure S2. Cytometry representative gate strategy and histogram from macrophages surface markers analysis.** Monocytes from healthy subjects were isolated from peripheral blood and cultured for 6 days in RPMI medium supplemented with 10% FBS and 50 ng/mL GM-CSF, and 5 μM decitabine or DMSO (0.2%) added for macrophage differentiation. On the 6^th^ day, differentiated macrophages were stimulated with cytokines for macrophage polarization or infected with Mtb for functional analysis. The M1 profile was defined by the addition of IFN-γ (100 ng/mL) and M2, by IL-4 and IL-13 (50 ng/mL each). M1 and M2 surface markers expression, CD86 and CD206, were analyzed by flow cytometry. Gate strategy and histograms are representative of different experimental conditions from one donor.
