# Supplementary figures and images for: Decitabine Promotes Modulation in Phenotype and Function of Monocytes and Macrophages That Drive Immune Response Regulation
Source: Cells. 2021 Apr 12;10(4):868. doi: 10.3390/cells10040868 (PMC8069756; doi:10.3390/cells10040868)

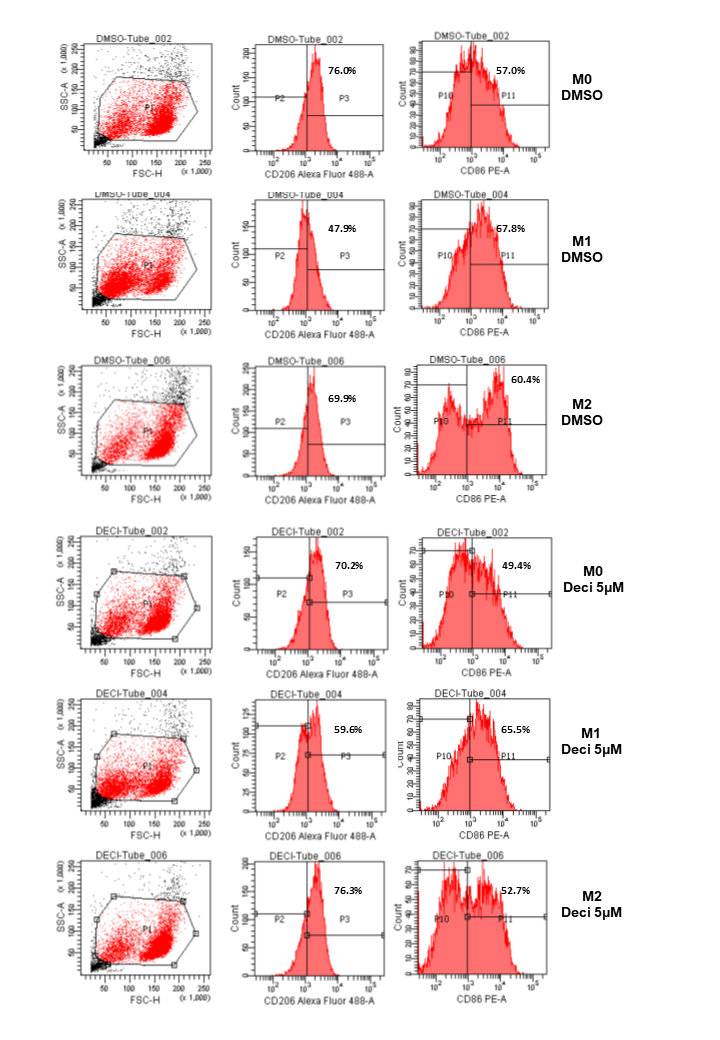

Supplement: Supplementary file 1 [file cells-10-00868-s001.zip › Sup_FAZ/Supplementary fig 2 - macrophages M1-M2 markers.tif]

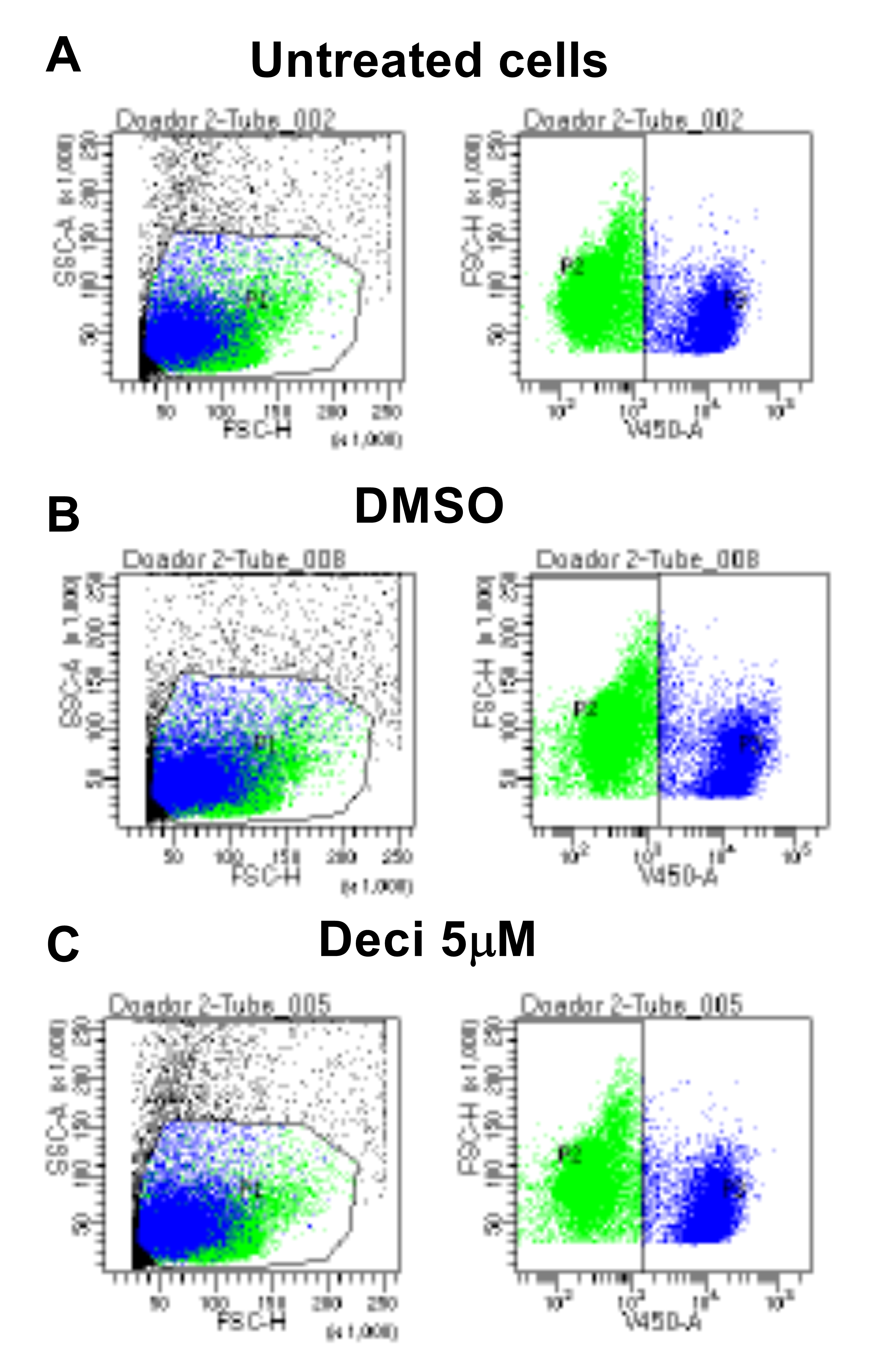

Supplement: Supplementary file 1 [file cells-10-00868-s001.zip › Sup_FAZ/Supplementary fig 1 - viability.tif]
